# Vasopressin Improves Cerebral Perfusion Pressure but Not Cerebral Blood Flow or Tissue Oxygenation in Patients with Subarachnoid Hemorrhage and Norepinephrine-Refractory Hypotension: A Preliminary Evaluation

**DOI:** 10.3390/jcm14238517

**Published:** 2025-12-01

**Authors:** Sylvia Bele, Elisabeth Bruendl, Nils Ole Schmidt, Martin Proescholdt, Martin Kieninger

**Affiliations:** 1 Department of Neurosurgery, Regensburg University Medical Center, 93052 Regensburg, Germanynils-ole.schmidt@ukr.de (N.O.S.); martin.proescholdt@ukr.de (M.P.); 2 Department of Anesthesiology, Regensburg University Medical Center, 93052 Regensburg, Germany

**Keywords:** subarachnoid hemorrhage, norepinephrine-refractory hypotension, vasopressin, cerebral blood flow, cerebral perfusion pressure, brain tissue oxygenation

## Abstract

**Background**: Maintaining an adequate mean arterial pressure (MAP) and cerebral perfusion pressure to ensure proper perfusion and oxygen delivery to all major organs is crucial—especially for neurosurgical patients after subarachnoid hemorrhage or traumatic brain injury—for preventing secondary brain damage or delayed cerebral ischemia. Currently, most neurosurgical intensive care units rely on intracranial pressure (ICP) and cerebral perfusion pressure (CPP) values to guide therapy. Fluid resuscitation and norepinephrine are standard treatments for achieving a CPP between 60 and 70 mmHg; however, patients sometimes experience norepinephrine-refractory hypotension. In such cases, vasopressin is often the preferred medication; it is widely utilized and has gained interest in treating septic shock or refractory hypotension following cardiac surgery or hypovolemic shock. Recent studies have also shown the significant impact of vasopressin on resuscitation after traumatic brain injury (TBI) and its effect on CPP during ICU care. Nevertheless, little is known about how vasopressin affects cerebral perfusion and oxygenation, especially in patients with subarachnoid hemorrhage. **Methods:** This preliminary retrospective single-arm study examined how vasopressin affects P_bt_O_2_ and cerebral blood flow using the non-invasive QuantixND^®^ device. After administering vasopressin for treating catecholamine-refractory hypotension, MAP, CPP, ICP, P_bt_O_2_, and cerebral blood flow were measured over a 20-min period. **Results:** In this small cohort, vasopressin sufficiently improved MAP and CPP over a 20 min period following AVP bolus administration with a slight decline at later time points. The ICP decreased throughout this period, indicating some level of autoregulation. In contrast, cerebral blood flow did not improve despite the rise in CPP, and P_bt_O_2_ levels remained below 20 mmHg. **Conclusions:** We conclude that vasopressin could be a viable option for maintaining MAP and CPP, but caution should be exercised in patients with already impaired cerebral perfusion. Furthermore, relying solely on CPP as the therapeutic guide in subarachnoid hemorrhage patients appears to be at least questionable.

## 1. Introduction

The primary goal of treatment for ICU patients is to maintain perfusion and oxygen delivery to all major organs to prevent organ damage. Since it is impossible to reverse the initial injury in neurosurgical patients, preventing further neuronal damage is essential. Therefore, avoiding early brain injury (EBI) following aneurysmal subarachnoid hemorrhage [1], raised intracranial pressure (ICP), or low cerebral perfusion pressure (CPP) due to low mean arterial blood pressure (MAP) after brain injury is critical. Especially in aSAH patients with cerebral vasospasm (CV), low CPP can lead to complications like delayed cerebral ischemia (DCI) or decreased tissue oxygenation [2]. Standard treatment guidelines aim to reduce the risk of brain ischemia, with a general CPP target above 70 mmHg often used in aSAH, though recommendations are still debated and are context-dependent [3]. In this study, we adopted a CPP of 70 mmHg as an institutional threshold despite the ongoing debate about specific thresholds. Managing CPP usually involves controlling ICP and MAP, since CPP = MAP − ICP. However, although many clinical trials have been conducted on this topic, there is no level I evidence defining an ideal CPP. Still, the Brain Trauma Foundation guidelines recommend a CPP between 50 and 70 mmHg for traumatic brain injury (TBI) patients, adjusted based on individual cerebral hemodynamics [4]. For patients with aneurysmal subarachnoid hemorrhage (aSAH), CPP requirements can vary significantly because the leading cause of death after aSAH is DCI, often resulting from cerebral vasospasm and increased vascular resistance [5,6]. A CPP > 70 mmHg should be maintained in those patients, or >100 mmHg if DCI occurs, as recommended in former studies [3]. The challenge with this guidance is that CPP is only an indicator of cerebral blood flow (CBF) since cerebral resistance is not incorporated into CPP values. Fluid resuscitation can help achieve the target CPP, but most patients also require vasopressors. Currently, catecholamines are preferred because they cannot cross the blood–brain barrier under normal conditions and can improve cerebral blood flow and oxygenation [7]. However, their use can cause side effects such as increased heart rate and myocardial oxygen consumption, especially at higher doses or with long-term use. These side effects may worsen extracranial complications of aSAH, including intravascular volume depletion or cardiac impairment, known as neurogenic myocardial injury (NMI) [8,9]. Increased systemic vascular resistance can also impair perfusion to vital organs [10,11]. Furthermore, some patients develop resistance to catecholamines [12], in which case arginine vasopressin (AVP) may be a valuable alternative.

Vasopressin, which plays a crucial role in maintaining osmotic and cardiovascular balance [13], is produced as a prehormone in the magnocellular neurons of the paraventricular and supraoptic nuclei in the hypothalamus. The hormone is cleaved into its active form and released into the bloodstream by the posterior pituitary gland. Arginine vasopressin interacts with various receptors, mainly AVPR1a (V1 receptor, mostly involved in vascular functions), AVPR1b (V3 receptor, primarily engaged in central functions), and AVPR2 (V2 receptor, mainly involved in renal functions). Additionally, AVP can also bind to oxytocin and purinergic receptors [14]. The V3 receptor is found in the hippocampus and anterior pituitary gland, and its stimulation by vasopressin causes the release of adrenocorticotropic hormone (ACTH), linking it to the corticosteroid axis in response to stressors such as hypotension [15,16].

The regulation of AVP’s vasoconstrictive effects involves a complex interaction of different actions and receptors. AVPR1a, a G protein-coupled receptor, is the primary effector for AVP-induced vasoconstriction and is located on vascular smooth muscle cells, platelets, and hepatocytes; for a review, see Russell 2019 [17]. It activates a phosphatidylinositol–calcium signaling pathway, which causes smooth muscle contraction [14]. Conversely, AVPR1a stimulation also increases the production of nitric oxide, a strong vasodilator in pulmonary [18] and coronary vessels [19]. Additionally, low doses of vasopressin that stimulate oxytocin receptors can lead to vasodilation [20].

A review of the literature by Russel in 2011 showed that, in patients with septic shock, using low-dose vasopressin combined with corticosteroids led to better outcomes than norepinephrine and steroids but also carried potential side effects such as peripheral ischemia or disturbances in microcirculation. Although studies have clearly demonstrated that AVP is safe to use and can have beneficial effects in septic shock [14,17] or in traumatic shock resuscitation when combined with low fluid resuscitation [10,21,22], validated recommendations for using AVP in either of these cases have yet to appear in clinical guidelines.

The use of AVP in neurosurgical and neurological patients remains highly debated. After a brain injury, vasopressin is released, which can trigger inflammatory reactions and cerebral edema [23]. Using AVPR1a antagonists has helped reduce secondary brain damage and swelling [24,25]. Interestingly, a model of blunt trauma to the head and chest showed that AVP was as effective as phenylephrine in maintaining CPP but was better at lowering ICP and improving cerebral tissue oxygenation [26]. In 2013, Van Haren et al. published a study on vasopressin’s role in managing CPP in patients with severe traumatic brain injury [27], finding that vasopressin is a safe and effective alternative to catecholamines for maintaining CPP. However, a study in humans found that vasopressin, despite preventing hypotension, reduced P_bt_O_2_ levels, increasing cerebral desaturation [28].

Since AVP’s vasoconstrictive potency is well known and the study by Van Haren et al. [27] did not measure cerebral blood flow (CBF) or brain tissue oxygenation (P_bt_O_2_), we aimed to examine whether the use of vasopressin is safe and how it affects MAP, CPP, ICP, P_bt_O_2_, and CBF in SAH patients with catecholamine-refractory hypotension.

The second aim of this preliminary study was to confirm if CPP alone is a sufficient parameter for therapy guidance in SAH patients.

## 2. Materials and Methods

### 2.1. Ethics/IRB Statement

This retrospective study was approved by the Local Ethics Committee at the University of Regensburg, Approval Number 24-3959-104. Informed consent for this study was waived due to the retrospective nature of data collection and the pseudonymization of the included patients.

All procedures involving human participants were carried out in accordance with the ethical standards of the institutional research committee and the 1975 Declaration of Helsinki, including its subsequent amendments.

All the above-mentioned interventions were not for study purposes and were performed strictly as therapeutic interventions. Written consent was obtained from all patients on whom the Quantix Doppler was used to evaluate the method, allowing the use of all data gathered during former research approved by the Local Ethics Committee at the University of Regensburg, Approval Number 18-1059-104.

### 2.2. Study Design

This is a preliminary, retrospective single-arm series with a 20-min observation window.

#### 2.2.1. Patient Selection and ICU Therapy

We primarily included patients treated for aSAH who already were undergoing P_bt_O_2_ monitoring, required vasopressin due to norepinephrine-refractory hypotension, and for whom we had sufficient time to use the Quantix. The location of the aneurysm causing the SAH and the severity of bleeding were documented according to the World Federation of Neurological Surgeons (WFNS) classification. All patients underwent continuous monitoring of ICP (Raumedic Neurovent^®^, Raumedic AG, Helmbrechts, Germany) and intra-arterial blood pressure to continuously calculate CPP. Additionally, in SAH patients, P_bt_O_2_ was measured using the Licox^®^ probe (Integra LifeSciences, Tullamore, Ireland) when patients were under analgesic sedation for more than 96 h or when CV was suspected based on elevated transcranial Doppler (TCD) values, according to our standard operating procedures [29]. Before vasopressin administration, the last transcranial Doppler (TCD) value was recorded to check for CV. CC was suspected if TCD velocities were >160 cm/s or showed an increase of >50% within 24 h. In addition, new neurological deficits with no other explanation were noted.

We used a fixed CPP threshold of >70 mmHg and/or P_bt_O_2_ above 20 mmHg as part of our local ICU standard operating procedure (SOP) for sedated, non-assessable aSAH patients or when cerebral vasospasm (CV) was suspected. Patients received volume and catecholamine therapy according to our SOP and recommendations based on the occurrence of cerebral vasospasm. Every SAH patient received 6 daily doses of 60 mg nimodipine via nasogastric tube for at least 96 h before vasopressin administration was initiated. The episode of refractory hypotension, which could not be adequately managed with volume, hydrocortisone, and catecholamines and led to vasopressin administration, was the first in every patient.

Also, patients were checked for digital ischemia and skin changes following AVP bolus administration.

In addition, we found 1 TBI patient (for demographics and measurement data, see Table 1 and Supplemental Data) who was treated according to the Brain Trauma Foundation guidelines [30] and those reported in the current literature [4]. Unfortunately, at the time of inclusion, P_bt_O_2_ monitoring for TBI patients was not standard in our ICU; thus, we included this patient only as a case vignette (see case vignette in Section 3).

#### 2.2.2. Therapeutic Intervention and CBF Monitoring

If CPP remained <70 mmHg under a norepinephrine dosage of >0.5 µg/kg/min and addition of epinephrine >0.15 µg/kg/min for 15 min, despite exhausting all options (volume and catecholamines) per our SOP, the attending ICU doctor decided to try AVP. A 3 IU bolus was administered, and the effects on MAP, ICP, CPP, P_bt_O_2,_ and CBF were monitored. The goal was to achieve a CPP above 70 mmHg and P_bt_O_2_ > 20 mmHg. Since this was a preliminary study, we measured CBF non-invasively using the Quantix ND device.

Ventilator settings, as well as sedation and analgesia, were not systematically adjusted during the 0–20 min observation period after AVP bolus administration, unless clinically necessary.

**Figure 1:**
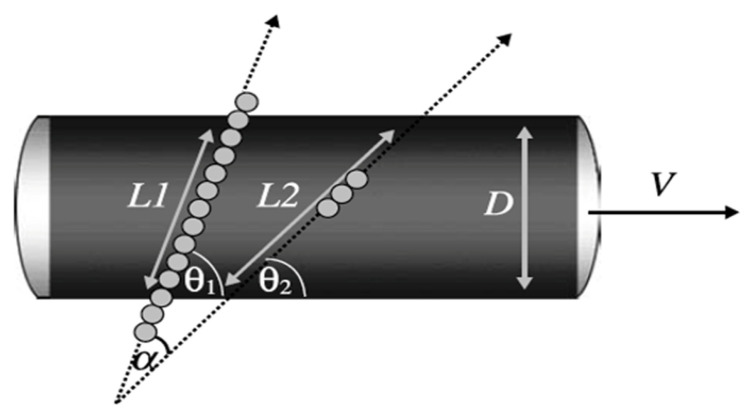
Principle of the extracranial volume flow measurement in the ACI, where α is the intonation angle. D = vessel diameter; V = volume flow; L1 and L2 = ultrasound beams; θ1 and θ2 = insonation angles of the ultrasound beams 1 and 2.

The Quantix ND (Cardiosonix Ltd., Ra’anana, Israel) uses an angle-independent Doppler method that employs two ultrasound heads positioned at specific angles relative to each other within a single insonation probe (Figure 1), projecting real-time flow diagrams onto a monitor. The probe was placed in the submandibular region of a patient lying on their back, and the internal carotid artery (ICA) was identified by its characteristic flow pattern shown on the monitor. On the real-time volume flow monitor, both angles (θ1, θ2), the measured vessel diameters (D1, D2), velocities consistent with laminar flow (L1, L2), and the shear force were observed (Figure 1). This information was continuously stored on a computer, allowing later replay and offline reevaluation. The system was tested at our clinic, which involved comparing the CBF measurements obtained with the Quantix ND to rCBF values [29,30,31,32].

We focused on measuring flow volume in the internal carotid artery (ICA), as previous studies showed that flow volume has an almost linear relationship with cerebral blood flow on the measured side. This correlation is even stronger when flow volume in both ICAs is measured and compared to overall CBF [31] (Figure 1).

When the decision to administer vasopressin was made, the last transcranial Doppler reading was recorded for all aSAH patients. The Quantix ND was used to establish the baseline flow volume and, therefore, the cerebral blood flow (CBF) before administering vasopressin. Additional measurements were taken at 2, 10, and 20 min after vasopressin administration; MAP, ICP, and CPP were also recorded at these time points.

#### 2.2.3. Flow Chart of Decision to Administer Vasopressin (Scheme 1)

Based on findings from our initial Quantix studies showing that Quantix values more accurately represented the risk of DCI than TCD values [32], the decision was made to change ventilator settings after vasopressin failed to improve CBF and P_bt_O_2_ after 20 min. A one-minute O_2_ flush with FiO_2_ of 1.0 was performed, followed by increasing the initial FiO_2_ to achieve PaO_2_ levels between 100 and 140 mmHg to enhance P_bt_O_2_. Additionally, we allowed hypercarbia up to PaCO_2_ of 50 mmHg to potentially dilate cerebral vessels. Because we could not rule out the influence of these changes in ventilator settings on CBF and P_bt_O_2,_ we decided to stop Quantix measurements.

In all patients, the temperature was maintained at or below 37.5 °C, serum sodium levels were kept between 135 and 145 mmol/L, and PaO_2_ was set at or above 75 mmHg unless CV occurred or PaO_2_ needed to be adjusted to 100–140 mmHg to ensure that P_bt_O_2_ exceeded 20 mmHg.

Patient outcomes were assessed at hospital discharge using the Glasgow Outcome Score (GOS), in which GOS 0 indicates death, GOS 1–3 indicates poor outcome, and GOS 4–5 indicates favorable outcome.

**Scheme 1:**
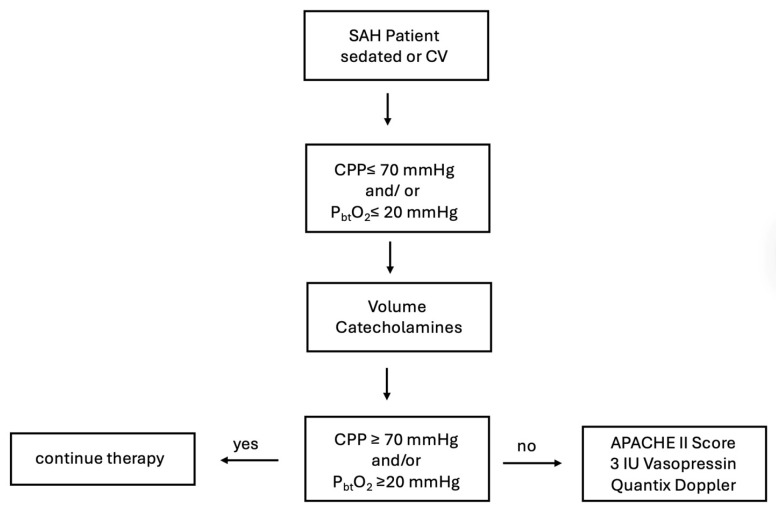
Flow Chart of Decision to Administer Vasopressin.

### 2.3. Statistical Analysis

Data were tested for normality using the Kolmogorov–Smirnov test. For descriptive statistics, results are presented as medians and ranges. Comparative statistical analysis evaluating the significance of differences in readouts of MAP, ICP, P_bt_O_2_, and CBF was performed using a repeated-measures ANOVA on ranks, which was employed as an exploratory within-subject test across time points following AVP bolus administration. Results with a *p*-value <0.05 were considered statistically significant but were interpreted descriptively due to the small number of patients. Analyses were conducted using Stata/IC (version 16.1, Stata Corp., College Station, TX, USA).

## 3. Results

Due to the small sample size, the single-arm design without a control group, and the 20-min observation window, the results of this study cannot be generalized.

Between 2012 and 2023, we reviewed 523 patient charts and identified 12 cases of aSAH with catecholamine-refractory hypotension. Four patients experienced vasoplegia due to nimodipine administration and were treated with methylene blue, while two patients lacked a P_bt_O_2_ probe and Quantix was not used to assess CBF. Thus, only six SAH patients met this study’s inclusion criteria, despite our hospital being a university facility with an annual caseload of 50–70 aSAH cases. Additionally, we found one patient treated for TBI in whom the Quantix Doppler was used; this patient was also included as a case vignette (see Table 1 for demographics). Four patients developed cerebral vasospasm. All but one patient received a 3 IU vasopressin bolus; patient 2 required an additional 3 IU bolus to improve their MAP. Regarding the Glasgow Outcome Scale (GOS), three patients had poor outcomes at hospital discharge (GOS 1–3), while two improved to GOS 4 after rehabilitation. Two patients died during hospitalization due to multi-infarct syndrome as a sequela of CV. For the detailed measurement data of each patient, see the Supplemental Data.

The APACHE II score increased before vasopressin administration, the PaO_2_ and PaCO_2_ levels were within normal limits, and body temperature and hemoglobin stayed in the normal ranges (see Table 2a).

We observed no pathological increases in the lactate (Table 2b) and blood glucose levels were in physiological ranges. Serum creatinine levels were normal in five patients and slightly elevated in one patient, who developed acute kidney injury four days after receiving an AVP bolus (see Table 2b). This patient required dialysis for five days but experienced good recovery of kidney function. The urine output ranged from 2810 mL to 5602 mL per 24 h. We are unable to provide the standard value in mL/kg/min because we could not determine the weight of three patients.

**Figure 2:**
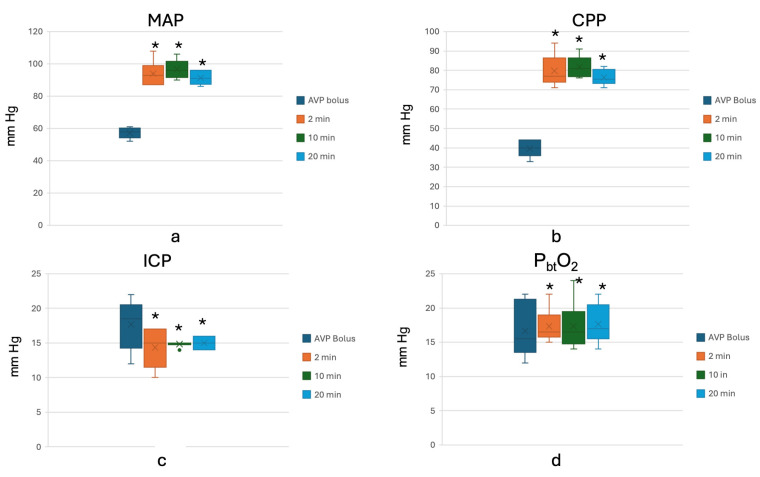
Box plot of examined data. (**a**–**d**) represent the changes in hemodynamics and brain oxygenation at AVP bolus administration and 2, 10, and 20 min following AVP administration in mmHg. * marks statistically significant changes within this small sample size.

**Figure 3:**
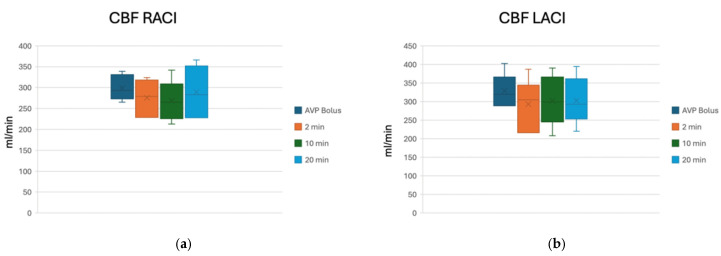
Box plot of CBF values. (**a**,**b**) show the results of the Quantix Doppler (CBF) at AVP bolus administration and at 2, 10, and 20 min after AVP administration in ml/min. RACI = right internal carotid artery; LACI = left internal carotid artery. No statistically significant changes in CBF were observed following AVP administration.

Two minutes after vasopressin administration, the MAP increased significantly and remained elevated throughout the observation period (*p* < 0.001). The MAP changes at TP 2–4 were statistically significant compared to the changes in TP 1, but no significant differences were observed among TP 2, 3, and 4, despite a decreasing trend over time (Table 3; Figure 2a). Similarly, CPP was significantly higher at TP 2–4 (*p* < 0.001), and this effect persisted for 20 min (Table 3, Figure 2b). Interestingly, the ICP values decreased in parallel with the MAP increase caused by vasopressin (*p* < 0.001; Table 3; Figure 2c), suggesting some intact autoregulation. The P_bt_O_2_ values initially stayed stable but increased significantly 10 min after vasopressin administration (*p* < 0.05; Table 3; Figure 2d). Despite this increase, P_bt_O_2_ never reached the target level of ≥20 mmHg. Lastly, CBF readings remained steady without significant changes for up to 20 min following vasopressin administration (*p* = 0.463; Table 3; Figure 3a,b).

### Case Vignette

One patient with severe TBI (GCS 7, see Table 1), rib fractures with lung contusions, and multiple bone fractures was also included in this study. Due to blood loss and the onset of pulmonary infection, catecholamine-refractory hypotension occurred 25 h after admission. After administering 3 IU of vasopressin, the patient demonstrated a sufficient rise in MAP and CPP, along with increased CBF. Consequently, the attending physician decided to continue vasopressin infusion at 0.03 IU/min for 12 h to maintain a CPP > 65 mmHg (see Supplemental Materials, patient 5, for details). Unfortunately, no P_bt_O_2_ probe was used, as this was not standard in TBI monitoring at that time. The patient was discharged after 23 days with a good outcome.

## 4. Discussion

In this retrospective study, we found that vasopressin (AVP) administration significantly increases MAP and CPP in neurocritical care patients with norepinephrine-refractory hypotension but does not improve CBF, especially in patients with already impaired cerebral perfusion. However, these changes must be considered to be within-subject in this small preliminary study.

Because the pathways of different AVP receptors are mostly independently regulated, synthetic agonists of these receptors are commonly used in modern medicine. For example, desmopressin, a V2 receptor agonist, is used to diagnose and treat diabetes insipidus and is also employed for various coagulopathies such as von Willebrand disease [33] or to counteract the effects of acetylsalicylic acid [21,34]. The AVPR1a agonist vasopressin (AVP), or terlipressin, is used in cases of postoperative bowel distention, refractory hypotension after cardiac surgery, intraoperative hypotension, or portal hypertension [35]. There has been a resurgence in AVP use in the treatment of hypovolemic [36] or septic shock and refractory hypotension, and is widely used for resuscitation [17,37].

Over the years, AVP has become increasingly relevant for treating vasoplegic septic shock and other refractory vasoplegic, catecholamine-resistant shocks [14,17]. AVP can restore vascular tone through at least four mechanisms—AVPR1a activation, NO modulation, ATP-sensitive K^+^ channel modulation, and enhancement of adrenergic vasoconstrictive agents—which cause small arterioles to contract and increase peripheral vascular resistance [38].

These various mechanisms suggest that AVP could be a potential option for use in neurosurgical patients. Although AVP can increase cerebral edema after ischemia [24,39] and may elevate rebleeding rates in an animal model of SAH [40], some studies have indicated that AVP is safe to use after TBI, with increases in MAP and CPP as endpoints [27,41], or in models where cerebral oxygenation was also assessed [26]. Animal studies have shown that AVP can positively affect CBF and cerebral oxygenation, but the results have been inconsistent [42]. A prospective study in humans under anesthesia found that AVP prevented hypotension but negatively impacted P_bt_O_2_, leading to increased cerebral desaturation [28]. To our knowledge, this preliminary study is the first to measure changes in MAP and CPP and focus on alterations in cerebral blood flow (CBF) and P_bt_O_2_ following AVP administration in SAH patients. In the small cohort included in our study, we observed a sufficient rise in MAP and CPP after administering 3 IU of AVP. Based on these findings, AVP appears to be a safe alternative vasopressor for neurosurgical patients, especially after TBI and aSAH, as it increases CPP. However, a concern arises from AVPR1a-mediated contraction of smooth muscle cells, which also seems to occur in cerebral vessels. This may explain the decrease or insufficient increase in CBF observed in our patients after AVP administration. This contrasts with norepinephrine, which mostly cannot cross the blood–brain barrier, raises systemic blood pressure without affecting cerebral vessels, and can increase CBF or metabolic oxygen intake, making it the most often used catecholamine for aSAH patients [7].

Those findings and our data raise two questions: first, whether CPP alone is truly the best target for treatment in TBI and aSAH patients, and second, whether vasopressin is safe for use in these patients.

Maintaining adequate MAP is crucial for all ICU patients, especially neurosurgical patients after SAH or TBI, to prevent delayed ischemia. Currently, most neuro ICUs rely on MAP, ICP, and CPP to direct treatment for these patients. According to the Brain Trauma Foundation guidelines, this approach remains appropriate, at least in TBI cases, but these simple treatment algorithms are increasingly being questioned [43,44]. After TBI, cerebral autoregulation may become impaired [45] and microcirculation often appears dysregulated, causing distended arterioles [46] and increased intracerebral blood volume, which can lead to a rise in ICP. Several studies recommend using a dynamic CPP approach in ICU care after TBI, tailored to each patient’s cerebrovascular autoregulatory capacity [44,47]. Depreitere and colleagues also found that CPP alone is insufficient for managing patients and incorporated the pressure reactivity index (PRx), developed by Czosnyka [48], into their monitoring protocol. While current evidence on P_bt_O_2_ use remains promising but mixed [49], two ongoing multicenter clinical trials—BOOST III and BONANZA—aim to more clearly define the role of P_bt_O_2_ monitoring in severe TBI. The completed multicenter OXY-TC trial [50,51] indicated that combining ICP and P_bt_O_2_ monitoring does not reduce the number of patients with poor neurological outcomes 6 months after TBI [50,51]. However, a pooled analysis and review by Chang et al. [52], following the OXY-TC trial, showed more encouraging results. Although the death rate was not significantly reduced by P_bt_O_2_-guided therapy, a much higher percentage of favorable outcomes was observed in the P_bt_O_2_-guided group. Therefore, more research is necessary to understand the significance of multimodal neuromonitoring in guiding treatment.

In our neurosurgical ICU, additional neuromonitoring techniques, such as P_bt_O_2_ and/or CBF, have been used alongside ICP and CPP to guide therapy after TBI since 2021 and in aSAH patients since 2014. However, this is not yet standard practice, as there is no level I evidence showing that ICP/CPP-guided therapy improves patient outcomes after TBI [53]. Findings by Van Haren et al. from 2013 suggest that disturbances in normal autoregulation, especially in peripheral arterioles and brain function after TBI, are complex and may affect multiple levels of function [27]. The effect of vasopressin on oxytocin receptors, causing vasodilation [14,17], is dose-dependently overridden by vasopressin-related AVPR1a activation, leading to vasoconstriction. This may explain the beneficial effects of vasopressin after TBI, as observed by Van Haren in 2013 and Dudkiewicz in 2008 [26]. AVPR1a activation increases MAP by constricting small cerebral vessels, reducing the intracranial blood volume, and lowering ICP. Our own study using data from a single TBI patient showed that AVP administration sufficiently increased MAP, reduced ICP, and increased CPP. From the concurrent increase in CBF, we infer that AVP can at least partially counteract autoregulatory dysfunction and optimize CPP and CBF without impairing CBF. Therefore, AVP appears to be safe for treating refractory hypotension in TBI patients and may even be more advantageous than other vasopressors because of its potential effects on small cerebral vessels. Norepinephrine can lower ICP only when autoregulation is intact, as it does not directly affect cerebral vessels because it cannot cross the blood–brain barrier under normal circumstances [7,54]. Thus, low-dose AVP might be a promising alternative to catecholamines. This hypothesis requires confirmation in larger studies for ICU management of TBI patients, especially when signs of impaired autoregulation are present. This is supported by Dudkiewicz and Proctor’s findings from 2008, which showed that AVP maintained CPP while improving ICP and cerebral tissue oxygenation more effectively compared to phenylephrine [26]. Additionally, a new multicenter trial is testing a drug that targets the impaired autoregulatory function of small, distended cerebral arterioles. The goal is for the drug to induce small vessel constriction, thereby lowering ICP.

The use of CPP as a therapeutic guide in patients with aSAH varies. Depending on the severity of the initial bleeding, patients may be awake and neurologically assessable or might be analgo-sedated and on a ventilator for extended periods. Cerebral vasospasm remains the primary cause of mortality and morbidity after aSAH: it decreases vessel diameter, increasing cerebrovascular resistance, and the resulting reduced blood flow is often followed by delayed cerebral ischemia, leading to severe neurological damage or death [55]. Guidance based on CPP after aSAH is challenging because only an approximation of CBF can be made, as it does not account for vessel diameter, resistance, or blood viscosity [3,56]. Nonetheless, increased vascular resistance significantly impacts CBF after aSAH. It is recommended to use brain oxygen measurements to prevent DCI caused by suboptimal CPP or CBF [57]. Studies have shown that P_bt_O_2_ values can help identify the optimal CPP, as patients with aSAH are at risk of inadequate CBF if CPP_opt_ is not achieved and may develop DCI.

We recommend using multimodal neuromonitoring, including P_bt_O_2_ (Licox^®^ Integra LifeScienes, Princeton, NJ, USA; or the Raumedic^®^ PTO probe; Raumedic AG, Helmbrechts, Germany) and, if available, CBF monitoring (Hemedex^®^ probe; Hemedex; St Waltham, MA, USA), along with ICP/CPP measurements to optimize ICU treatment and determine the appropriate MAP and CPP for each patient. This approach may reduce volume load and catecholamine use by enabling an ideal MAP level to be set for each patient (typically between 70 and 140 mmHg). In aSAH, we routinely monitor P_bt_O_2_ and CBF and have achieved good outcomes [29,58], but so far there is no level I evidence that additional neuromonitoring significantly improves patient outcomes [59]. Although MMM-guided therapy often yields better results, the lack of evidence is not only due to limited studies but also the heterogeneity of treatment SOPs across hospitals and the variability in individual patient needs [60], which makes it challenging to implement standardized treatment protocols reliably across all clinics. Establishing standardized interventions is essential before proceeding with complex outcome studies using MMM-guided treatment.

Maintaining adequate CPP, based on dependent variables such as P_bt_O_2_ and CBF, is typically achieved through hypertension therapy [5]. When catecholamine-refractory hypotension occurs, AVP may again be the preferred drug due to its vasoconstrictive effects. However, our data suggest that increasing MAP, accompanied by decreased ICP and higher CPP, did not necessarily lead to a corresponding improvement in CBF in aSAH patients. Additionally, we observed no increase in P_bt_O_2_ values following vasopressin administration, especially in patients with elevated TCD velocities indicative of cerebral vasospasm. This could be related to AVP’s dose-dependent effects; administering a 3 IU bolus of vasopressin might stimulate AVPR1a, potentially overriding the beneficial effects of oxytocin receptor activation and increased NO release in the brain. Modulating NO release to promote vasodilation could be an effective strategy to prevent severe cerebral vasospasm after SAH. Recent studies suggest that cerebral vasospasm is not solely a disease of larger vessels but may also occur early in the microcirculation. Terpolilli et al. presented promising data on the use of NO ventilation to prevent microcirculatory vasospasm after aSAH (presented at the 2014 annual DGNC meeting). Because AVP can also influence and release NO, it might be advantageous in the early stages after aSAH to prevent vasospastic events in the microcirculation. Nonetheless, further research is necessary to explore this potential application.

Our preliminary data indicate that AVP can counteract norepinephrine-refractory hypotension in SAH patients but does not necessarily improve CBF. In three cases, CBF decreased even after AVP administration despite significant increases in CPP, suggesting that AVP may exert vasoconstrictive effects on cerebral blood vessels. This implies that, while AVP may help with refractory hypotension, its use should be approached with caution in patients with impaired cerebral perfusion. More comprehensive studies involving continuous invasive CBF monitoring and larger patient samples are needed to establish the definitive role of AVP in these scenarios.

## 5. Conclusions

Our conclusion involves two hypotheses. First, AVP is an alternative vasopressor used to treat refractory hypotension and prevent organ hypoperfusion in ICU patients. It seems feasible for TBI patients, but our initial data suggest that, overall, caution should be exercised when using it in neurosurgical patients, especially when CBF could be compromised, e.g., by cerebral vasospasm, since it does not necessarily improve CBF or P_bt_O_2_ levels in aSAH patients. When administering AVP to these patients, CBF or P_bt_O_2_ should be monitored to determine whether the rise in CPP also increases brain oxygenation, which could help prevent cerebral ischemia. In addition, safety measures such as monitoring creatinine and urine output or changes in skin color should be taken to detect AVP’s extracranial effects early on.

Secondly, our data, in line with the current literature [3], indicate that relying solely on CPP to guide therapy may not be sufficient, especially for patients with aSAH and sequelae such as cerebral vasospasm, since vascular tone is not considered in the CPP formula. Also, the use of a “one size fits all” target CPP should be reassessed, as discussed herein, to reach an optimal CPP for each patient. We were unable to confirm an “optimal” CPP in this study because we did not use individualized CPP thresholds for the patients evaluated.

## 6. Shortcomings

The present preliminary study has several limitations that may affect data interpretation. First, the small number of patients and thus the limited statistical power reduce the significance of the results and the data analysis, so the conclusions cannot be generalized. The second issue is that it is a retrospective single-arm study without a control group, which certainly introduces bias. Additionally, we did not individualize CPP (e.g., CPP opt), so we cannot determine an ideal CPP value.

The third point of critique could be the brief measurement duration (just 20 min after AVP administration), which limits the ability to observe AVP’s effects at later time points.

Larger prospective studies involving more patients, with measurements of cerebral tissue oxygenation and CBF using intraparenchymal probes like the QFlow 500^TM^ by Hemedex, Waldham, MA, USA^®^, should be conducted to validate these findings.

## Figures and Tables

**Table 1:** Patient demographics.

	Gender	Age	Diagnosis	WFNS	GCS	AL	Vasospasm	GOS
Patient 1	f	57	aSAH	2	13	MCA	y	1
Patient 2	m	43	aSAH	4	8	ACoA	n	5
Patient 3	f	48	aSAH	3	11	MCA	y	3
Patient 4	m	61	aSAH	1	15	ACoA	y	1
Patient 5	m	40	TBI	na	7	na	na	4
Patient 6	f	42	aSAH	3	9	MCA	y	3
Patient 7	f	58	aSAH	2	13	ACoA	n	3

Table presents demographic data for the 7 patients. AL = aneurysm location; MCA = middle cerebral artery; ACoA = anterior communicating artery; na = not applicable; GCS = Glasgow Coma Scale; GOS = Glasgow Outcome Scale; aSAH = aneurysmal subarachnoid hemorrhage; TBI = traumatic brain injury; WFNS = World Federation of Neurosurgical Societies Score.

**Table 2:** (**a**) Safety measures → patient demographics and blood gas measurements. (**b**): Safety measures → blood gas measurements.

(**a**)								
	**APAII**	**APA II x**	**Temp.**	**Hb**	**PaO_2_**	**PaO_2_x**	**PaCO_2_**	**PaCO_2_x**
Patient 1	5	9	37.1	10.4	96	90	45	38
Patient 2	6	11	36.9	9.2	102	78	35	39
Patient 3	8	12	37.0	8.6	92	83	32	38
Patient 4	5	12	37.4	9.8	105	86	41	42
Patient 5	8	14	36.1	9.6	94	74	35	37
Patient 6	6	14	36.8	9.8	98	90	39	35
(**b**)								
	**Creatinine 1** **mg/dL**		**Creatinine 2** **mg/dL**		**Sodium** **mmol/L**		**Lactate 1** **mg/dL**	**Lactate 2** **Mg/dL**
Patient 1	0.88		1.02		138		9	14
Patient 2	0.69		1.15		142		10	15
Patient 3	1.01		1.2		135		12	18
Patient 4	0.66		0.93		141		5	17
Patient 5	0.79		1.02		136		7	18
Patient 6	1.23		1.43		142		10	16

(**a**) presents additional patient data from the six SAH patients. APA II = APACHE II score on admission; APAIIx = APACHE II score closest to vasopressin administration; Hb = hemoglobin in g/dL; PaO_2_ and PaCO_2_ = values in mmHg at time of admission or following intubation; PaO_2_x and PaCO_2_x = values closest to AVP bolus administration; Temp = body temperature in °C. (**b**) shows the following data: Creatinine 1 = levels on admission (norm 0.67–1.17 mg/dL); Creatinine 2 = value on day of AVP bolus administration; sodium = level in blood gas closest to AVP bolus administration; Lactate 1 = lactate level on admission; Lactate 2 = lactate in blood gas closest to AVP bolus administration.

**Table 3:** Median values of MAP, CPP, ICP, P_bt_O_2_, and CBF measured by Quantix.

	MAP Median	Range	CPP Median	Range	ICP Median	Range	P_bt_O_2_ Median	Range	LACI Median	RACI Median
TP1	58	52/61	40	37/44	18.5	12/22	15.5	12/22	319	293
TP2	93 *	87/108	77 *	71/94	15 *	10/17	16.5 *	15/22	305	279
TP3	96 *	90/106	81 *	76/91	15 *	14/15	16.5 *	14/24	299	264
TP4	91 *	86/96	75.5 *	71/82	14.5 *	12/16	17 *	14/22	293	283

Table shows all measured parameters (median and range = 1% and 99% percentile in mmHg). MAP = mean arterial pressure in mmHg; CPP = cerebral perfusion pressure in mmHg; ICP = intracranial pressure in mmHg; P_bt_O_2_ = cerebral oxygenation in mmHg; LACI = cerebral blood flow on the left side in mL/min; RACI = cerebral blood flow on the right side in mL/min. Values marked with * are statistically significant.

## Data Availability

The original contributions presented in the study are included in the article (and Supplementary Materials), further inquiries can be directed to the corresponding authors.

## Supplementary Materials

The following supporting information can be downloaded at: https://www.mdpi.com/article/10.3390/jcm14238517/s1.

## Abbreviations

The following abbreviations have been used and were clarified within the text:

aSAH	aneurysmal subarachnoid hemorrhage
CPP	cerebral perfusion pressure
CPP_opt_	optimal cerebral perfusion pressure
CBF	cerebral blood flow
MAP	mean arterial pressure
ICP	intracranial pressure
P_bt_O_2_	brain tissue oxygenation
TBI	traumatic brain injury
AVP	arginin-vasopressin or terlipressin
AVPR	arginin-vasopressin receptor
PRx	pressure reactivity index
ICU	intensive care unit
CV	cerebral vasospasm
TCD	transcranial Doppler
GCS	Glasgow Coma Score
GOS	Glasgow Outcome Score
IU	international units
ICA	internal carotid artery
RMCA	right middle cerebral artery
LMCA	left middle cerebral artery
ACoA	anterior communicating artery
TP	time point
EBI	early brain injury
DCI	delayed cerebral ischemia
NMI	neurogenic myocardial injury
ACTH	adrenocorticotropic hormone
