# Supplementary material for: Vasopressin Improves Cerebral Perfusion Pressure but Not Cerebral Blood Flow or Tissue Oxygenation in Patients with Subarachnoid Hemorrhage and Norepinephrine-Refractory Hypotension: A Preliminary Evaluation
Source: J Clin Med. 2025 Dec 1;14(23):8517. doi: 10.3390/jcm14238517 (PMC12693331; doi:10.3390/jcm14238517)
Supplement: Supplementary file 1 [file jcm-14-08517-s001.zip › jcm-3951912-supplementary.pdf]

Supplemental Data

		Supplemental Data				Quantix ml/min		TCD cm/sec	
**Patient**	**TP**	**MAP**	**ICP**	**CPP**	**PbtO2**	**LACI**	RACI	LMCA	RMCA
	1	56	12	44	22	289	276		
	2	94	10	84	18	216	229	217	198
	3	92	15	77	16	208	213		
1	4	86	15	71	18	220	228		
	mean	82	13	69	18,5	233,25	236,5		
	1	52	15	37	16	354	339		
	2	87	12	75	22	330	316	132	128
	3	90	14	76	24	358	342		
2	4	88	14	74	22	350	347		
	mean	79,25	13,75	65,5	21	348	336		
	1	60	20	40	21	289	276		
	2	87	16	71	16	216	229		
	3	94	15	79	18	258	232	210	188
3	4	90	15	75	20	278	266		
	mean	82,75	16,5	66,25	18,75	260,25	250,75		
	1	61	17	44	14	326	265		
	2	108	14	94	15	302	246	165	176
	3	106	15	91	14	288	230		
4	4	96	14	82	16	264	228		
	mean	92,75	15	77,75	14,75	295	242,25		
	1	52	18	35	n.a.	387	341	n.a.	n.a.
	2	99	13	86	n.a.	442	386		
	3	98	12	86	n.a.	428	392		
5	4	89	12	77	n.a.	402	366		
	mean	84,5	13,75	71		414,75	371,25		
	1	60	20	40	15	312	310		
	2	92	17	75	17	308	312	172	130
	3	100	15	85	17	310	298		
6	4	92	16	76	16	308	366		
	mean	86	17	69	16,25	309,5	321,5		
	1	55	22	33	12	402	328		
	2	96	17	79	15	387	324	143	165
	3	98	15	83	15	390	298		
7	4	96	16	80	14	394	300		
	mean	86,25	17,5	61,25	14	393,25	312,5		

Supplemental data show the measurement results of each individual patient. TP= time points; MAP= mean arterial pressure; ICP= intracranial pressure; CPP= cerebral perfusion pressure; $P_{bt}O_2$= brain tissue oxygenation; ACIl= left internal carotid artery; ACI r= right internal carotid artery; LMCA= left medial cerebral artery; RMCA= right medial cerebral artery, TCD= transcranial Doppler values in cm/s.
